# Feasibility work to inform the design of a randomized clinical trial of wound dressings in elective and unplanned abdominal surgery

**DOI:** 10.1002/bjs.10274

**Published:** 2016-08-04

**Authors:** Severn and Peninsula Audit and Research Collaborative for Surgeons (SPARCS) and West Midlands Research Collaborative (WMRC) on behalf of the Bluebelle Study Group, L. Andronis, M. Calvert, L. Magill, J. Mathers, T. D. Pinkney, A. Torrance, H. Talbot, J. M. Blazeby, N. S. Blencowe, J. Coast, T. Draycott, J. Donovan, R. Gooberman‐Hill, B. C. Reeves, C. A. Rogers, R. Longman, M. Woodward, T. Young, J. Bird, G. Clayton, L. Ellis, R. Macefield, T. Milne, H. van der Nelson, A. Nicholson, L. Rooshenas, D. Siassakos, S. Strong, D. Townsend, C. McMullan, C. Winter, G. Atherton, H. Tafazal, A. Eriksson, T. Chapman, Z. Zafar, J. Chang, E. Sharma, N. Green, U. Shariff, T. Neito, H. Youssef, P. Marriott, M. Popplewell, N. Ring, A. Sharples, V. Summerour, A. Bhangu, E. Upchurch, T. Hardy, J. Monteiro de Barros, L. Reza, C. Ekere, A. Greenwood, S. Strong, C. Florance, P. Orchard, E. Court, C. Ives, E. Papworth, C. Lee, S. Buchan, J. Bennett, C. Rowlands, L. Frank, K.‐A. Ide, E. Noble, H. Sellars, E. Anderson, R. Fallaize, J. Kynaston, E. Hotton, J. Banks, N. Thompson, T. Hodkinson, N. Blencowe, R. Bamford, P. Newman, J. Cutting, Z. Barber, C. Grant, J. Mason, J. Bailey

**Affiliations:** ^1^ University of BirminghamBirmingham; ^2^ University of BristolBristol; ^3^ University Hospitals Bristol NHS Foundation TrustBristol; ^4^ Welsh Wound Innovation Centre, University of CardiffCardiff; ^5^ University of BristolBristol; ^6^ University of BirminghamBirmingham; ^7^ North Bristol NHS TrustBristol; ^8^ Warwick HospitalWarwick; ^9^ Royal Stoke HospitalStoke; ^10^ New Cross HospitalWolverhampton; ^11^ Good Hope HospitalSutton Coldfield; ^12^ Queen Elizabeth HospitalBirmingham; ^13^ Heartlands HospitalBirmingham; ^14^ Sandwell HospitalWest Bromwich; ^15^ Cheltenham General HospitalCheltenham; ^16^ Derriford HospitalPlymouth; ^17^ Gloucester Royal InfirmaryGloucester; ^18^ Great Western HospitalSwindon; ^19^ Musgrove Park HospitalTaunton; ^20^ North Bristol NHS TrustBristol; ^21^ North Devon District HospitalBarnstaple; ^22^ Royal Devon and Exeter HospitalExeter; ^23^ Royal United HospitalBath; ^24^ Torbay HospitalTorbay; ^25^ University Hospitals Bristol NHS Foundation TrustBristol; ^26^ Weston General HospitalWeston‐super‐Mare; ^27^ Yeovil District HospitalYeovil

## Abstract

**Background:**

Designing RCTs in surgery requires consideration of existing evidence, stakeholders' views and emerging interventions, to ensure that research questions are relevant to patients, surgeons and the health service. When there is uncertainty about RCT design, feasibility work is recommended. This study aimed to assess how feasibility work could inform the design of a future pilot study and RCT (Bluebelle, HTA ‐ 12/200/04).

**Methods:**

This was a prospective survey of dressings used to cover abdominal wounds. Surgical trainees from 25 hospitals were invited to participate. Information on patient risk factors, operation type and type of wound dressings used was recorded for elective and unplanned abdominal procedures over a 2‐week interval. The types of dressing used were summarized, and associations with operation type and patient risk factors explored.

**Results:**

Twenty hospitals participated, providing data from 727 patients (1794 wounds). Wounds were predominantly covered with basic dressings (1203 of 1769, 68·0 per cent) and tissue adhesive was used in 27·4 per cent (485 of 1769); dressing type was missing for 25 wounds. Just 3·6 per cent of wounds (63 of 1769) did not have a dressing applied at the end of the procedure. There was no evidence of an association between type of dressing used and patient risk factors, type of operation, or elective and unscheduled surgery.

**Conclusion:**

Based on the findings from this large study of current practice, the pilot study design has evolved. The inclusion criteria have expanded to encompass patients undergoing unscheduled surgery, and tissue adhesive as a dressing will be evaluated as an additional intervention group. Collaborative methods are recommended to inform the design of RCTs in surgery, helping to ensure they are relevant to current practice.

## Abstract 2

Relevant background work

## Introduction

Dressings are used widely to cover wounds at the end of surgical procedures; in some specialized areas (such as paediatric surgery) they are not applied routinely. This may reflect the different ways that approaches to treatment are adopted in clinical practice, or the lack of evidence to suggest that dressings confer any benefit^1^, ^2^. A Cochrane systematic review summarizing evidence for the use of dressings to prevent surgical‐site infection (SSI) was published in 2011^3^ and updated in 2014^4^. Twenty RCTs were included, which examined different types of dressing and no dressing on a closed wound. All trials were assessed as having an unclear or high risk of bias and were underpowered to detect SSI events. No evidence was identified to suggest that any dressing significantly reduced the risk of developing an SSI compared with leaving wounds exposed; neither was there any benefit associated with particular dressing types. The review concluded that decision‐making around dressings may need to be informed by cost and practical issues surrounding symptom management. It also recommended that the design of future RCTs should focus on surgical procedures at highest risk of an SSI, such as abdominal surgery, and evaluate the dressings that health professionals use most widely.

The uncertainties raised in this review led the National Institute for Health Research (NIHR) Health Technology Assessment (HTA) to identify wound dressings as a research area likely to make a substantial difference to people's health. Research was commissioned to examine whether an RCT in this area would be possible and the Bluebelle pilot study (HTA ‐ 12/200/04) was funded to address this question^5^. If deemed possible, the main trial will investigate which type(s) of dressing reduce the risk of SSI among patients undergoing abdominal surgery.

One current area of uncertainty facing surgical RCTs is selecting which interventions to evaluate. This requires consideration of existing evidence, current practice and emerging novel interventions to ensure that the RCT findings would be relevant to patients, surgeons and the health service. There are many different wound dressings available, ranging from basic to advanced with varying absorbent, adherent and interactional properties^6^. The NIHR HTA commissioned call highlighted the need to justify which interventions should be evaluated. This study, therefore, aimed to understand and characterize the use of perioperative abdominal wound dressings in current practice, to inform the design of the future study.

## Methods

A prospective multicentre study was undertaken by members of the Severn and Peninsula Audit and Research Collaborative for Surgeons (SPARCS)^7^ and the West Midlands Research Collaborative (WMRC)^8^. All hospitals within the two trainee‐led research collaborative networks were invited to participate, via e‐mails and personal communication. A surgical trainee‐level principal investigator, responsible for local coordination of data collection and entry, was identified within each participating hospital. The study was registered with the clinical audit department in each hospital and approval was obtained for the Bluebelle study from the National Research Ethics Service (14/LO/0640, Camden and Islington, 10 April 2014).

Abdominal wounds created during elective or unplanned abdominal surgery, and closed primarily, were surveyed during a 2‐week interval in January 2015. A wound was considered to be closed primarily if the edges of incised skin were opposed (using suture material, tissue adhesive or clips) at the end of the procedure. Vascular, gynaecological, urological and paediatric procedures were excluded. Cases were included only if trainees were present (and therefore able to collect the data prospectively). Trainees completed anonymized data collection forms at the end of each surgical procedure, recording information about skin closure and dressings (*Appendix S1*, supporting information). Dressings were categorized as advanced (those with advanced practical and/or therapeutic properties, including amorphous material, silicone, hydrocolloid, foam, antimicrobials or negative pressure) or basic (those without advanced or therapeutic properties which are adherent around the perimeter or entire surface, with or without a pad to absorb exudate). ‘No dressing’ was documented when an already closed wound was left without a covering at the end of the operation. Use of tissue adhesive to cover an already closed wound (whereby it was used as a dressing rather than wound closure technique) was categorized separately.

Operative and patient‐related risk factors that might influence dressing selection were recorded. Operative risk factors included the type of procedure performed and access (open, laparoscopic or laparoscopically assisted), whether a stoma was formed, and the degree of wound contamination (clean, clean‐contaminated, contaminated and dirty)^9^. Procedures were classified as planned (elective) or unplanned (emergency). The following patient‐related risk factors were recorded: age, sex, BMI, diabetic status and ASA fitness grade.

The reason for dressing selection (by the surgeon responsible for closing the wound) was recorded in the following three categories: personal preference, selected for specific wound characteristics, or that the dressing was simply handed to the surgeon at the end of the procedure, without discussion. Dressings could be selected for multiple reasons and space was provided for free‐text answers. To supplement this, procurement officers from each hospital were contacted to obtain information about local policies for purchasing dressings.

### Data management and analysis

Data were entered into a password‐protected online database held on a server (developed and maintained by the Bristol Clinical Trials and Evaluation Unit) in one of the participating hospitals. Analyses were performed in Stata^®^ version 13 (StataCorp, College Station, Texas, USA) and summarized the frequency of different dressing types using descriptive statistics. Descriptive statistics were also used to examine whether patient characteristics or the type and urgency of surgery were associated with particular dressing strategies.

## Results

In total, 25 hospitals within the SPARCS and WMRC networks were approached and 20 participated. Data from 727 patients (1794 wounds) were included, of whom 193 (26·5 per cent) underwent upper gastrointestinal surgery (*Table* 1). The number of wounds per patient varied from one to seven: one in 299 patients (41·1 per cent), two in 51 (7·0 per cent), three in 155 (21·3 per cent), four in 190 (26·1 per cent), five in 25 (3·4 per cent), and just seven patients (1·0 per cent) had more than five wounds. Complete data sets were submitted for 675 patients (92·8 per cent). There was one missing data item for 36 patients (5·0 per cent) and 16 (2·2 per cent) had more than one missing item.

**Table 1:** Descriptive data for patients and procedures

	No. of patients
	(*n* = 727)
Age (years)*	
< 30	119 (16·5)
30–40	90 (12·4)
41–50	104 (14·4)
51–60	109 (15·1)
61–70	144 (19·9)
> 70	157 (21·7)
Sex ratio (M : F)*	348 : 375
ASA fitness grade†	
I	224 (31·1)
II	342 (47·4)
III	140 (19·4)
IV	15 (2·1)
Diabetes‡	
No	659 (91·3)
Insulin‐dependent	51 (7·1)
Non‐insulin‐dependent	12 (1·7)
BMI (kg/m^2^)§	
< 20	50 (7·1)
20–25	276 (39·1)
26–30	237 (33·6)
> 30	142 (20·1)
Surgical procedure	
Upper gastrointestinal surgery	
Oesophagogastric resection	8 (1·1)
Pancreaticobiliary resection	11 (1·5)
Antireflux surgery	10 (1·4)
Bariatric surgery	11 (1·5)
Cholecystectomy	153 (21·0)
Lower gastrointestinal surgery	
Colectomy	82 (11·3)
Hartmann's procedure	10 (1·4)
Rectal resection	40 (5·5)
Stoma formation	24 (3·3)
Stoma closure	24 (3·3)
General surgery	
Groin hernia repair	90 (12·4)
Abdominal wall hernia repair	38 (5·2)
Appendicectomy	109 (15·0)
Laparoscopy/laparotomy	81 (11·1)
Small bowel resection	9 (1·2)
Adhesiolysis	8 (1·1)
Other	19 (2·6)

Values in parentheses are percentages. Data missing for

* four,

† six,

‡ five and

§ 22 patients.

Sutures were most commonly used to achieve skin closure (1531, 86·5 per cent), with clips (158, 8·9 per cent) and Steri‐Strips™ (3M, St Paul, Minnesota, USA) (48, 2·7 per cent) used less commonly. Of the 1794 wounds, dressing type was recorded for 1769, with 1706 (96·4 per cent) covered and 63 (3·6 per cent) not covered by a dressing. The majority of dressings were classified as basic (1203 of 1769, 68·0 per cent), with just 1·0 per cent (18 of 1769) advanced. Tissue adhesive was applied over closed skin to 27·4 per cent of wounds (485 of 1769).

### Use of dressings according to operative and patient risk factors

Variation in the types of dressing according to the category, urgency and type of surgery is described in *Tables* 2 and 3. Dressing types were similar across different types of procedure, and between elective and unscheduled surgery. There was no apparent association between the type of dressing used and patient risk factors, such as diabetes, stoma formation, BMI and ASA fitness grade.

**Table 2:** Dressing types according to operative factors

	Basic		Advanced		Tissue adhesive		No dressing	
	Patients	Wounds	Patients	Wounds	Patients	Wounds	Patients	Wounds
	(*n* = 512)	(*n* = 1203)	(*n* = 17)	(*n* = 18)	(*n* = 186)	(*n* = 485)	(*n* = 31)	(*n* = 63)
Operation category								
Clean	199 (38·9)	449 (37·3)	2 (12)	2 (11)	58 (31·2)	128 (26·4)	11 (35)	24 (38)
Clean‐contaminated	242 (47·3)	606 (50·4)*	12 (71)	13 (72)	106 (57·0)	305 (62·9)	14 (45)	33 (52)
Contaminated	50 (9·8)	115 (9·6)	2 (12)	2 (11)	12 (6·5)	32 (6·6)	5 (16)	5 (8)
Dirty	21 (4·1)	33 (2·7)	1 (6)	1 (6)	10 (5·4)	20 (4·1)	1 (3)	1 (2)
Urgency of surgery†								
Elective	320 (62·6)	809 (67·3)	10 (59)	11 (61)	132 (71·0)	371 (76·5)	22 (71)	51 (81)
Emergency	191 (37·4)	393 (32·7)	7 (41)	7 (39)	54 (29·0)	114 (23·5)	9 (29)	12 (19)
Surgical approach								
Laparoscopic	223 (44·6)	756 (62·8)	5 (29)	5 (28)	102 (54·8)	353 (72·8)	16 (52)	42 (67)
Open	228 (44·5)	243 (20·2)	7 (41)	8 (44)	61 (32·8)	63 (13·0)	9 (29)	10 (16)
Mixed	61 (11·9)	204 (17·0)	5 (29)	5 (28)	23 (12·4)	69 (14·2)	6 (19)	11 (17)
Type of operation								
Upper gastrointestinal	132 (25·8)	465 (38·7)	1 (6)	1 (6)	55 (29·6)	211 (43·5)	7 (23)	22 (35)
Lower gastrointestinal	119 (23·2)	256 (21·3)	11 (65)	12 (67)	54 (29·0)	122 (25·2)	7 (23)	17 (27)
General	261 (51·0)	482 (40·1)	5 (29)	5 (28)	77 (41·4)	152 (31·3)	17 (55)	24 (38)

Values in parentheses are percentages. The total number of patients across all dressing groups is 746 (not 727) as some patients had different types of dressing applied and therefore fell into more than one category. This table does not include the 25 wounds for which dressing type was not recorded.

* Interpret as: there were 606 clean‐contaminated wounds in 242 of 512 patients in the basic dressing group.

† Information missing for one wound [1 patient] (basic dressing category).

**Table 3:** Dressing types according to risk factors

	Basic		Advanced		Tissue adhesive		No dressing	
	Patients	Wounds	Patients	Wounds	Patients	Wounds	Patients	Wounds
	(*n* = 512)	(*n* = 1203)	(*n* = 17)	(*n* = 18)	(*n* = 186)	(*n* = 485)	(*n* = 31)	(*n* = 63)
Stoma formation	56 (10·9)	96 (8·0)	5 (29)	5 (28)	32 (17·2)	70 (14·4)	6 (19)	9 (14)
Diabetes*	43 (8·4)	85 (7·1)	2 (13)	2 (14)	17 (9·2)	51 (10·5)	3 (10)	6 (10)
ASA fitness grade†								
I	163 (32·0)	403 (33·7)	5 (29)	6 (33)	55 (29·7)	148 (31)	8 (27)	20 (32)
II	238 (46·8)	584 (48·8)	7 (41)	7 (39)	92 (49·7)	231 (48)	16 (53)	31 (50)
III	98 (19·3)	198 (16·7)	5 (29)	5 (28)	36 (19·5)	96 (20)	6 (20)	11 (18)
IV	10 (2·0)	11 (0·9)	0 (0)	0 (0)	2 (1·1)	6 (1)	0 (0)	0 (0)
BMI (kg/m^2^)‡								
< 20	36 (7·3)	81 (7·0)	1 (6)	1 (6)	12 (6·5)	19 (4·0)	3 (11)	9 (15)
20–25	196 (39·5)	426 (36·9)	5 (31)	5 (29)	74 (40·0)	175 (36·4)	13 (46)	23 (39)
26–30	163 (32·9)	401 (34·7)	6 (38)	7 (41)	63 (34·1)	165 (34·3)	8 (29)	19 (32)
> 30	101 (20·4)	246 (21·3)	4 (25)	4 (24)	36 (19·5)	122 (25·4)	4 (14)	8 (14)

Values in parentheses are percentages. The total number of patients across all dressing groups is 746 (not 727) as some patients had different types of dressing applied and therefore fell into more than one category. This table does not include the 25 wounds for which dressing type was not recorded. Information missing for:

* eight wounds [3 patients] (4 [2] basic, 4 [1] advanced);

† 12 wounds [4 patients] (7 [3] basic, 4 [1] tissue adhesive, 1 [1] no dressing – 1 patient had 2 different wound dressings); ‡58 wounds [20 patients] (49 [16] basic, 1 [1] advanced, 4 [1] tissue adhesive, 4 [3] no dressing – 1 patient had 2 different wound dressings).

### Reasons for selection of dressings

Most surgeons used the dressings that were handed to them by the nursing staff at the end of the operation (925, 76·5 per cent) (*Table* 4). Information from 29 procurement staff revealed that cost was the overwhelming factor when selecting which dressings to purchase, enabling bulk ordering and keeping the range of available dressings to a minimum.

**Table 4:** Reasons for dressing selection, according to type of dressing

	Basic		Advanced	
	Patients	Wounds	Patients	Wounds
	(*n* = 512)	(*n* = 1203)	(*n* = 17)	(*n* = 18)
Handed by nursing staff†	380 (75·1)	909 (76·3)	15 (88)	16 (89)
Personal preference‡	170 (33·6)	371 (31·2)	1 (6)	1 (6)
Wound characteristics§	53 (10·5)	120 (10·1)	5 (29)	5 (28)
Other*, ¶	4 (0·8)	10 (0·8)	0 (0)	0 (0)

Values in parentheses are percentages. Some patients had different types of dressing applied and therefore fell into more than one category. Dressings could be selected for multiple reasons and so totals can add up to more than 100 per cent.

* Common reasons included: standard practice and to keep the wound waterproof to allow showering. Information missing for:

† 12 wounds [6 patients] (all basic dressings);

‡ 12 wounds [6 patients] (all basic dressings);

§ ten wounds [5 patients] (all basic dressings);

¶ 13 wounds [7 patients] (12 [6] basic, 1 [1] advanced).

## Discussion

This multicentre study has described the use of perioperative wound dressings in elective and unplanned abdominal surgery across two regions of the UK. A total of 727 patients (1794 wounds) were studied over 2 weeks and data completeness was very high (92·8 per cent). Of the covered wounds, basic wound dressings were used mainly (68·0 per cent) and advanced dressings applied rarely (1·0 per cent). Unexpectedly, tissue adhesive (which had not been included in either basic or advanced categories) was used as a dressing in 27·4 per cent of wounds. Dressing types were similar across different types of procedure, and between elective and unplanned surgery, and were not influenced by patient or operative risk factors. Surgeons typically used the dressings handed to them by nursing staff (according to local hospital policy) rather than favouring one particular type, even when patients were high risk (severely obese or diabetic). These findings have important implications for the design of a main RCT. They highlight the need to evaluate tissue adhesive as a separate trial group, and to increase the inclusion criteria to encompass patients undergoing unscheduled as well as elective surgery.

Pretrial work is seen increasingly as crucial to the success of RCTs, and may be particularly relevant to complex interventions such as surgery^10^. Recommendations for good practice in the design of pretrial work highlight several opportunities to reduce uncertainty^11^. These include estimating the size of the eligible population and recruitment rates, developing and selecting outcome measures, estimation of parameters required for sample size calculations and determining the acceptability of interventions. The design of some studies may expose further uncertainties such as specifying the most appropriate interventions or eligibility criteria. One way of resolving these uncertainties is to study current practice in a representative sample, which may be challenging in complex environments such as the operating theatre. Trainee surgeons have formed research collaboratives as a novel solution to undertaking multicentre surgical studies^12^, ^13^, ^14^. In the present study, complete data sets were submitted for 92·8 per cent of patients, demonstrating the potential for trainees to generate large amounts of high‐quality data that are directly relevant to an RCT.

Specific strengths of this study are the contemporaneous collection of prospective data across multiple operating theatres in different hospitals with very few missing fields, and the inclusion of elective and unplanned abdominal surgery. Despite this, some weaknesses remain. It is possible that some eligible patients were not captured during the study, meaning that variations in practice may have been missed, although the large sample size from 20 different centres means that this is unlikely. A further limitation is that data were collected from two distinct geographical regions and it is possible that findings are not representative of the entire UK.

This study, undertaken by surgeons and methodologists, demonstrates the importance of collaboration and teamwork to ensure how information can be obtained efficiently to inform trial design. The finding that tissue adhesive was used widely as a dressing was unexpected. Currently, only four RCTs^15^, ^16^, ^17^, ^18^ have evaluated tissue adhesive as a dressing, none of which included patients undergoing gastrointestinal surgery. Additionally, they are small, single‐centre studies and each has aspects of their design that were subject to a high risk of bias. There is, therefore, a need for this product to be evaluated fully in a pragmatic trial to generate high‐quality evidence to inform practice.

Based on the findings of the present study, the pilot study design has evolved. First, the inclusion criteria will be expanded to encompass patients undergoing unscheduled as well as elective surgery. Second, three groups (tissue adhesive as a dressing *versus* a basic dressing *versus* no dressing) rather than two groups (basic dressing *versus* no dressing) will be evaluated. Inclusion of the no‐dressing group is important because of a lack of evidence to support the use of dressings^3^, ^4^ and because not applying dressings to closed wounds is common in paediatric practice. Whether it is possible to randomize patients into an RCT with a no‐dressing group, and whether patients and staff can comply with treatment allocations, is unknown. These uncertainties justify the need for a pilot study before a definitive multicentre RCT. As well as collecting data about SSI (the proposed primary outcome), the pilot study will collect information on secondary measures such as practical wound management issues, cosmesis and cost‐effectiveness.

The successful design and conduct of RCTs in surgery can be optimized by appropriate, high‐quality pretrial work. Although such work has traditionally focused on recruitment, outcome assessment and completeness of follow‐up data, it is also critical to identify the appropriate interventions to evaluate, especially in the context of surgical RCTs.

## Collaborators

Core study group: N. S. Blencowe, J. M. Blazeby, S. Strong (Centre for Surgical Research, School of Social and Community Medicine, University of Bristol, Division of Surgery, Head and Neck, University Hospitals Bristol NHS Foundation Trust, and Bristol Surgical Trials Centre, Bristol); A. Torrance, T. D. Pinkney (Academic Department of Surgery, School of Cancer Sciences, Queen Elizabeth Hospital, University of Birmingham, and Birmingham Surgical Trials Centre, Birmingham); G. Clayton, L. Ellis, H. Talbot, B. C. Reeves, C. A. Rogers (Clinical Trials and Evaluation Unit, School of Clinical Sciences, University of Bristol, Bristol). This study was conceptualized by N.S.B., J.M.B., A.T., T.D.P., B.C.R. and C.A.R. Data collection was coordinated by N.S.B., A.T., L.E. and H.T. Data analysis was performed by G.C., N.S.B. and C.A.R. N.S.B. wrote the first draft of the manuscript, which was edited by all members of the core study group. J.M.B. is the chief investigator of the Bluebelle study and the guarantor for this paper.

Members of the Bluebelle Study Group contributed to the study design and contributed to paper writing. Co‐applicants: L. Andronis, M. Calvert, L. Magill, J. Mathers, T. D. Pinkney, A. Torrance (University of Birmingham, Birmingham); J. M. Blazeby, N. S. Blencowe, J. Coast, T. Draycott, J. Donovan, R. Gooberman‐Hill, B. C. Reeves C. A. Rogers, (University of Bristol, Bristol); R. Longman, M. Woodward (University Hospitals Bristol NHS Foundation Trust, Bristol); T. Young (Welsh Wound Innovation Centre, University of Cardiff, Cardiff). Other members of the Bluebelle study management group: J. Bird, G. Clayton, L. Ellis, R. Macefield, T. Milne, H. van der Nelson, A. Nicholson, L. Rooshenas, D. Siassakos, S. Strong, D. Townsend (University of Bristol, Bristol); C. McMullan (University of Birmingham, Birmingham); C. Winter (North Bristol NHS Trust, Bristol).

The following trainees collected data from hospitals across the West Midlands (WMRC) and South West of England (SPARCS). WMRC: G. Atherton, H. Tafazal, A. Eriksson (Warwick Hospital, Warwick); T. Chapman, Z. Zafar (Royal Stoke Hospital, Stoke); J. Chang, E. Sharma (New Cross Hospital, Wolverhampton); N. Green, U. Shariff, T. Neito, H. Youssef (Good Hope Hospital, Sutton Coldfield); P. Marriott (Queen Elizabeth Hospital, Birmingham); M. Popplewell, N. Ring, A. Sharples (Heartlands Hospital, Birmingham); V. Summerour, A. Bhangu (Sandwell Hospital, West Bromwich). SPARCS: E. Upchurch (Cheltenham General Hospital, Cheltenham); T. Hardy, J. Monteiro de Barros, L. Reza, C. Ekere (Derriford Hospital, Plymouth); A. Greenwood, S. Strong, C. Florance (Gloucester Royal Infirmary, Gloucester); P. Orchard, E. Court (Great Western Hospital, Swindon); C. Ives, E. Papworth, C. Lee, S. Buchan (Musgrove Park Hospital, Taunton); J. Bennett, C. Rowlands (North Bristol NHS Trust, Bristol); L. Frank, K.‐A. Ide (North Devon District Hospital, Barnstaple); E. Noble, H. Sellars, E. Anderson (Royal Devon and Exeter Hospital, Exeter); R. Fallaize, J. Kynaston, E. Hotton (Royal United Hospital, Bath); J. Banks, N. Thompson, T. Hodkinson (Torbay Hospital, Torbay); N. Blencowe, R. Bamford, P. Newman (University Hospitals Bristol NHS Foundation Trust, Bristol); J. Cutting, Z. Barber, C. Grant (Weston General Hospital, Weston‐super‐Mare); J. Mason, J. Bailey (Yeovil District Hospital, Yeovil).

**Supporting information**

Additional supporting information may be found in the online version of this article:

**Appendix S1** Data collection pro forma (Word document)

## Editor's comments

This is a valuable study, and the final RCT will be more relevant as a consequence. The biggest surprise is that surgeons seem to be very accepting of the dressing they are handed by the scrub nurse. Although this might be a consequence of hospital purchasing policy, surgeons are not normally so passive, so to me it suggests they are not convinced by any existing evidence that operative dressing type makes much difference. That is why the planned study is so important, and surgeons should be encouraged to participate, and to include a no‐dressing group.

J. Earnshaw

*Editor, BJS*

## Supporting information


**Appendix S1** Data collection pro forma
