# Supplementary material for: Feasibility work to inform the design of a randomized clinical trial of wound dressings in elective and unplanned abdominal surgery
Source: Br J Surg. 2016 Aug 4;103(12):1738–44. doi: 10.1002/bjs.10274 (PMC5091629; doi:10.1002/bjs.10274)
Supplement: Supplementary file 1 — Appendix S1 Data collection pro forma [file BJS-103-1738-s001.docx]

# **Feasibility work to inform the design of a randomized clinical trial of wound dressings in elective and unplanned abdominal surgery**

Severn and Peninsula Audit and Research Collaborative for Surgeons (SPARCS) and West Midlands Research Collaborative (WMRC) on behalf of the Bluebelle study group

# **Appendix S1** Data collection pro forma
